# Iloperidone and Temozolomide Synergistically Inhibit Growth, Migration and Enhance Apoptosis in Glioblastoma Cells

**DOI:** 10.3390/biomedicines12061134

**Published:** 2024-05-21

**Authors:** Sahar Mubeen, Iffat Raza, Badaruddin Ujjan, Bushra Wasim, Lubna Khan, Nadia Naeem, Syed Ather Enam, Farina Hanif

**Affiliations:** 1 Department of Anatomy, Dow International Medical College, Dow University of Health Sciences, Karachi 75330, Pakistan; sahar.mubeen@duhs.edu.pk; 2 Department of Anatomy, Karachi Institute of Medical Sciences, Karachi 75080, Pakistan; dr.iffat@kimsmalir.edu.pk; 3 Department of Neurosurgery, Dow University Hospital, Dow University of Health Sciences, Karachi 74200, Pakistan; badaruddin.ujjan@duhs.edu.pk; 4 Department of Anatomy, Ziauddin University Hospital, Karachi 75600, Pakistan; bushra.wasim@zu.edu.pk; 5 Department of Biochemistry, Dow International Medical College, Dow University of Health Sciences, Karachi 75330, Pakistan; lubnakhanku@yahoo.com; 6 Dow Research Institute of Biotechnology & Biomedical Sciences, Karachi 75330, Pakistan; nadia.naeem@duhs.edu.pk; 7 Center of Oncological Research in Surgery, Aga Khan University Hospital, Karachi 74800, Pakistan; ather.enam@aku.edu

**Keywords:** glioblastoma, iloperidone, temozolomide, atypical anti-psychotic

## Abstract

Glioblastoma (GBM) is a fatal astrocytic glioma with poor prognosis and treatment resistance. Repurposing potential FDA-approved drugs like anti-psychotics can address the concerns in a timely and cost-effective manner. Epidemiological studies have shown that patients with schizophrenic using anti-psychotics have a low incidence of GBM. Therefore, we aimed to investigate the therapeutic potential of atypical anti-psychotic Iloperidone (ILO) alone and in combination with Temozolomide (TMZ) against GBM. The study assessed the growth inhibitory effect of ILO, TMZ, and their combination (ILO + TMZ) on U-87MG and T-98G cell lines using an MTT assay. The drug interaction coefficient (CDI) was determined, and doses with synergistic effects were used for subsequent experiments, including migratory, invasion, and TUNEL assays. The expressions of DRD2, β-catenin, Dvl2, Twist, and Slug were assessed by RTq-PCR, whereas the β-catenin protein expression was also determined by immunocytochemistry. ILO (*p* < 0.05) and TMZ (*p* < 0.01) significantly inhibited the growth of U-87MG cells at all tested doses. The combination of 60 µM of both drugs showed synergistic activity with CDI < 1. The inhibition of migration and apoptosis was more pronounced in the case of combination treatment (*p* < 0.001). Inhibition of the invading cells was also found to be significant in ILO- and combination-treated groups (*p* < 0.001). ILO and combination treatment also significantly downregulated the expression of DRD2, while TMZ upregulated the expression (*p* < 0.001). The expressions of β-catenin (*p* < 0.001), Dvl2 (*p* < 0.001), Twist (*p* < 0.001), and Slug (*p* < 0.001) were also significantly downregulated in all treatment groups as compared to the vehicle control. The data suggest that ILO possesses strong growth inhibitory activity, possibly due to its effect on DRD2 and β-catenin expression and has the potential to be repurposed against GBM.

## 1. Introduction

Glioblastoma (GBM) is a fatal heterogeneous astrocytic glioma which is considered invincible to date. Rapid growth, an infiltrative tendency, and the capacity to swiftly penetrate neighboring brain tissue are characteristics of GBM. Glioblastoma has the stigma of poor prognosis and recurrence, which can be attributed to its resistance to conventional treatments, which include surgical resection followed by chemotherapy with Temozolomide (TMZ) and radiation [1,2]. TMZ has been used as a primary DNA methylating agent and causes cell cycle arrest at the G2/M phase. No other drugs have been found to be as efficient as TMZ in terms of cytotoxicity. However, GBM cells with high levels of DNA repair mechanisms can potentially reverse the methylation effects of TMZ, leading to resistance against TMZ therapy. This leads to poor response and survival in 50% of patients who receive TMZ as primary chemotherapy due to TMZ resistance in GBM [3,4,5].

A fraction of stem-like GBM cells, genetic heterogeneity, TMZ resistance, and poor penetrance of drugs into the tumor due to the blood–brain barrier (BBB) are the obstacles to the development of successful GBM treatments [6]. The average survival rate of individuals with GBM has not improved despite recent developments in biology and the therapeutics of the disease. Keeping in view the situation, the repurposing of potential drugs is warranted, as this strategy is cost- and time-effective. The repurposing of anti-psychotics like Perphenazine, Haloperidol, Clozapine, Fluspirilene, and Penfluridol has already shown potential against various cancers, including GBM [7,8,9]. Also, various epidemiological studies have highlighted that GBM incidence is lower in patients with schizophrenia taking anti-psychotics [5,6,7]. Most of the anti-psychotics target the Dopamine receptor 2 (DRD2), which has been shown to be overexpressed in GBM and is crucial for tumor development and progression [9,10,11,12,13,14]. Anti-psychotics’ easy penetrance through the BBB is an added advantage [11,14]. Studies have documented that typical anti-psychotic, such as chlorpromazine, clozapine, and risperidone, can lower the risk of developing numerous malignancies like breast, lung, and colorectal cancer and GBM [7,15,16]. However, in comparison to typical anti-psychotics, atypical anti-psychotics are considered to be much safer because they have less extrapyramidal side effects, better tolerance, and hence, a lower risk of death [15]. So far, only a few atypical anti-psychotics, like olanzapine, clozapine, and risperidone, have been investigated in GBM, and they have been shown to possess moderate anti-tumor activity [17,18,19]. Still, many atypical anti-psychotics are underexplored or even unexplored for their activity against GBM. This rationalizes that unstudied atypical anti-psychotics should be investigated in GBM to discover the best available options for the repurposing of these drugs against GBM.

Further, the wingless (Wnt)/β-catenin pathway has been found to be deregulated in various malignancies and has also been reported as a culprit in GBM pathogenesis [18,19,20]. The pathway is switched on when Wnt ligands bind to Frizzled and Low-density lipoprotein receptor-related proteins. The binding leads to the recruitment of disheveled (Dvl2) phosphoprotein at the plasma membrane, initiating a series of events that disrupts the β-catenin destruction complex [APC (adenomatosis polyposis coli) /Axin/ glycogen synthase kinase (GSK3β)], which leads to stabilization of β-catenin and hence, accumulation in cytoplasm and translocation into the nucleus [21,22,23]. It has been claimed that the pathway is already activated in cancers, and TMZ further induces activation of the pathway, leading to chemoresistance [24,25,26]. Wnt/β-catenin signaling also regulates downstream transcription factors like Twist 1/2, Zeb1/2, and Snail/Slug [27,28]. Twist is a transcription factor of the basic helix–loop–helix class that is implicated in the upregulation of β-catenin expression [29], tumor initiation, progression, and metastasis by promoting epithelial-to-mesenchymal transition (EMT). It is characterized by the loss of cell–cell adhesion and the acquisition of migratory and invasive properties leading to therapeutic resistance [22,30,31]. Upregulated Twist expression has been reported in GBM, and it has also been suggested as a driver for the EMT-regulating gene Slug [32,33]. Wnt/β-catenin activation also upregulates Slug expression, which has been shown to inhibit the epithelial phenotype of numerous cancer cell types and increase the migration and invasion of malignant gliomas [17,26,30,34,35]. 

Recent studies have reported that anti-psychotics modulate the Wnt/β-catenin pathway and therefore may have a role in limiting cancer progression [1,7,8,15]. It has also been found that DRD2 antagonists inhibit the Wnt pathway by modulating GSK-3β and Dvl2, thus limiting cancer progression [8,36]. 

Keeping in view the lacuna of research related to the available atypical anti-psychotics and their potential against malignancies with an edge in terms of minimal side effects and effective penetrance via BBB, the present study aims to explore the anti-tumor activity of Iloperidone (ILO) alone and in combination with TMZ against U-87 MG GBM cells. ILO is a new-generation atypical anti-psychotic agent and an FDA-approved atypical anti-psychotic that antagonizes DRD2 and serotonin receptors. Chemically, ILO is a benzisoxazole like risperidone and shows a multiple-receptor-binding profile, sharing this feature with the other atypical anti-psychotic agents [37]. The present study reports the effects of the DRD2 antagonist (ILO) and standard chemo-therapeutic drug TMZ as a monotherapy and in combination on GBM cell proliferation, migration, apoptosis, and Wnt/β-catenin pathway genes. We aimed to discover if TMZ’s anticancer efficacy could be considerably enhanced by ILO against GBM. To the best of our knowledge, no comprehensive study has yet been carried out to explore the effect of ILO on GBM at the molecular level.

## 2. Materials and Methods

### 2.1. Cell Line and Reagents

For this in vitro experimental study, human GBM cell lines U-87 MG and T-98 G were obtained from the American Type Tissue Culture Collection (ATCC, Manassas, VA, USA). Iloperidone (ILO) was purchased from Chem-Impex International (Wood Dale, IL, USA). Temozolomide (TMZ), Trypsin, paraformaldehyde, 3-(4,5-dimethylthi-azol-2-yl)-2,5-diphenyltetrazolium bromide (MTT), and Triton X-100 were purchased from Sigma-Aldrich, St. Louis, MO, USA. Dulbecco’s Modified Eagle medium (DMEM), penicillin/streptomycin, and fetal bovine serum (FBS) were purchased from Gibco (Waltham, MA, USA). Total RNA isolation kit, cDNA synthesis kit, Power Up SYBR Green Master Mix, rabbit-anti β-catenin antibody, Alexa-Flour^®^546 anti-rabbit IgG, and 4′,6-diamidino-2-phenylindole (DAPI) were bought from ThermoFisher Scientific, Waltham, MA, USA. The Dead End TUNEL Assay system kit was purchased from Promega Co-operation, Madison, Wisconsin, WI, USA.

### 2.2. Stock Preparation of the Drugs

Different concentrations of ILO and TMZ (20 µM, 40 µM, 60 µM, and 80 µM) were used for the treatment. Multiple combination doses of ILO and TMZ were tried and tested in order to select the most effective combination dose at which cell growth inhibition was synergistic. The doses were selected considering previous studies where IC_50_ of 80 µM was reported for ILO; a range of 10 µM to 100 µM was used for TMZ [38,39,40,41]. ILO and TMZ were dissolved in 100% sterile DMSO at a stock concentration of 50 mM and carefully stored at −20 °C until further use. A working solution of the drugs and compounds was freshly prepared by diluting a stock solution in the culture medium. The final concentration of DMSO did not exceed 0.1% (*v*/*v*) in the culture medium. DMSO was used as a vehicle control (VC). Before each experiment, ILO and TMZ were diluted from the stock solution to the final concentration with DMEM. All experiments were conducted in triplicate and repeated independently at least 3 times.

### 2.3. Cell Culture

U-87 MG and T-98G cell lines were cultured as monolayers in T-25 flasks. U-87 MG cells were maintained in DMEM High Glucose supplemented with 10% FBS, 1% penicillin/streptomycin, 1% Amphotericin, 1% L-Glutamine, and 1% sodium Pyruvate. T-98G cells were maintained in α-MEM, 2 mM Glutamine, 1% Non-Essential Amino Acids, 1% Sodium Pyruvate, and 10% of fetal bovine serum (FBS). The cultures were maintained at 37 °C in a humidified atmosphere containing 5% CO_2_. 

### 2.4. 3-(4,-5-Dimethyl-thiazol-2-yl)-2,5-diphenyl-tetrazolium Bromide (MTT) Assay

An MTT assay was carried out to analyze the growth inhibitory activity of ILO and TMZ on the U-87 MG cell line. The assay was conducted in triplicates. For this analysis, GBM cells were plated (5 × 10^3^ cells/100 µL) in 96-well plates and incubated at 37 °C in 5% CO_2_. Once the monolayer was established, the cells were treated with ILO and TMZ at different concentrations (20 µM, 40 µM, 60 µM, and 80 µM) for 48 h. Following the 48 h treatment period, 200 µL of MTT (0.5 mg/mL) was added in each well and incubated for 3 h. MTT dye was then discarded, and 100 µL of DMSO was added to the cells. An ELISA reader was used to record the absorbance at 490 nm of the wavelength. The growth inhibition percentage was computed using the standard formula given below [42]:

Percentage Growth Inhibition of cells = 100 − [(At − Ab)/(Ac − Ab)] × 100

where At stands for the absorbance value of the test compound, Ab for blank, and Ac for control.

### 2.5. Synergy Analysis of the Test Drugs

In order to determine whether the growth inhibitory activity of TMZ is enhanced by ILO, U-87 MG, and T-98G, the cells were treated with various combinations of these drugs for 48 h, and the MTT assay was performed. The coefficient of drug interaction (CDI) method was used to compute the combinatorial effect of ILO and TMZ against GBM cells’ proliferation. A CDI value less than, equal to, or greater than 1 specifies that the drugs are synergistic, additive, or antagonistic, respectively. The formula used is [CDI = AB/ (A × B)], where AB represents the ratio of combination drugs to VC, and A and B represent the ratio of individual drugs to the VC group [37]. The minimal doses (60 µM for both ILO and TMZ) that produced a synergistic effect were used in the rest of the experiments.

### 2.6. Migration Assay 

This in vitro technique was used to assess the effects of ILO, TMZ, and their combination on the migratory potential of cells in 2 dimensions. Briefly, the cells were plated in 6-well plates. Upon achieving confluency, a cell-free area was created by physically making a scratch with a 10 µL pipette tip. After the scratch, cells were treated with VC, ILO (60 µM), TMZ (60 µM), and a combination of both drugs. The wound closure area was observed via imaging the scratch area after 48 h at 4× magnification. The images were taken from both treated and VC groups, followed by quantification of the closure area using ImageJ software (Version 1.8.0). 

### 2.7. Invasion Assay

The Transwell invasion assay was performed to check the effect of ILO, TMZ, and their combination on the inhibition of the invasive potential of U-87 MG cells. Briefly, 150,000 cells were plated per well on a 6-well plate, and the cells’ monolayer was treated with the specified drugs dosage. After 48 h, the cells were resuspended in media containing 0.1% FBS and seeded 100,000 cells in chambers pre-coated with 0.1% gelatin in triplicates. Cells that migrated/invaded and appeared on the bottom surface of the Transwell insert membrane were fixed with 75% methanol and stained with crystal violet, followed by washing with distilled water. Representative pictures of the cells were acquired at 10× magnification, and the total number of invasive cells on random fields was counted and analyzed using ImageJ software (Version 1.8.0) [43]. 

### 2.8. Terminal Deoxynucleotidyl Transferase dUTP Nick End Labeling (TUNEL) Assay 

DNA fragmentation represents a characteristic hallmark of apoptosis, and TUNEL is an established method used for detecting DNA fragmentation that represents late-stage apoptosis. The Dead End Colorimetric TUNEL system kit (Promega, Corporation) end-labels the fragmented DNA of apoptotic cells that help in the identification of cells undergoing apoptosis. Briefly, 2 × 10^5^ cells were plated in a 24-well plate and treated with ILO (60 μM), TMZ (60 μM), and their combination. The cells were then washed with PBS and fixed with 4% paraformaldehyde, followed by permeabilization with 0.2% Triton. Later, the fixed cells were incubated using the TUNEL reagent mix (equilibrium buffer, biotinylated nucleotides, Terminal Deoxynucleotidyl Transferase, and Recombinant rTDT enzyme) at 37 °C for 1 h. This was followed by the addition of the binding agent Horseradish peroxidase-labeled streptavidin (Streptavidin HRP) for 30 min. Finally, the chromogen diaminobenzidine (DAB) was added until the background appeared light brown. The cells labeled as dark brown nuclei were considered apoptotic. Quantification of apoptotic cells was carried out by counting the total cells and apoptotic cells in five blindly selected fields. The apoptotic index of the cells was calculated as per the following formula [44]:

Apoptotic index (%) = (number of apoptotic cells/total number of cells) × 100

### 2.9. Gene Expression Analysis via RTq PCR

The effect of the drugs on the expression level of DRD2 and Wnt/β catenin pathway-related genes (β-catenin, Dvl2, Twist, and Slug) was analyzed using real-time PCR. RNA from GBM cells, treated with either ILO (60 µM), TMZ (60 µM), or their combination, was extracted using a total RNA isolation kit after 48 h. RNA integrity and purity were determined using nanodrop and gel electrophoresis, respectively. c-DNA was then synthesized and subjected to RTq-PCR using Power Up syber green master mix and specific primers sequences (Table S1) against DRD2, β-catenin, Twist, Dvl2, and Slug. The expression of the housekeeping gene (β-Actin) was used to normalize the expression of other genes. The fold change in the relative expression of each target mRNA was calculated based on the comparative CT (2^−∆∆CT^) method. 

### 2.10. Immunocytochemistry 

Immunocytochemistry was carried out to analyze the protein expression of β-catenin in drug-treated and VC cells. For the experiment, cells were plated in chamber slides in triplicates, and once the monolayer was established, they were treated with ILO, TMZ, and their combination at 37 °C and 5% CO_2_. Following the 48 h treatment, cells were fixed with 4% paraformaldehyde, washed with Phosphate-Buffered Saline (PBS), and blocked with a blocking solution at 37 °C for 1 h. Next, cells were washed with PBS again and incubated with the anti-rabbit β-catenin antibody at 4 °C overnight. The next day, the cells were washed with PBS and then incubated for 1 h at room temperature with secondary antibody Alexa-Flour^®^546 anti-rabbit IgG in PBS. The cells were then washed again and counterstained with 4′,6-diamidino-2-phenylindole (DAPI). The stained slides were then mounted with PBS + glycerol and visualized under a fluorescent microscope. Quantification studies of the images were carried out using ImageJ software (Version 1.8.0) [45].

### 2.11. Statistical Analysis

The data are expressed as mean ± SEM and were analyzed using GraphPad Prism 8 and SPSS 20. One-way analysis of variance (ANOVA) was used to analyze differences between groups, followed by a post hoc (Bonferroni) test. A *p*-value of <0.05 was considered statistically significant. 

## 3. Results

### 3.1. ILO and TMZ Have Growth Inhibitory Potential against U-87 MG GBM Cells

**Figure 1:**
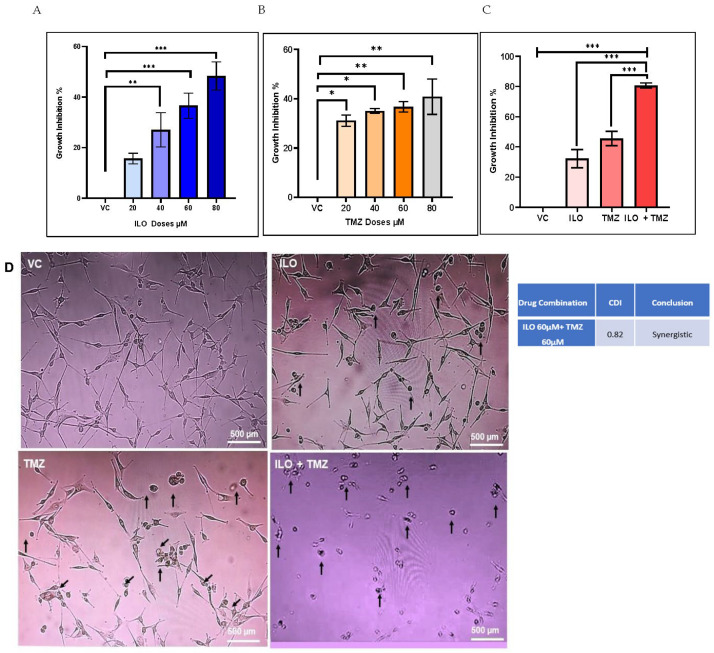
The graphical illustrations of the effects of ILO (**A**), TMZ (**B**), and their combination (**C**) on U-87 MG growth. The cells were treated with different doses of ILO and TMZ (20 µM, 40 µM, 60 µM, and 80 µM) for 48 h, followed by an MTT assay. Bar graph illustrating dose-dependent growth inhibition of U-87 MG cells with ILO and dose-independent growth inhibition of U-87 MG cells with TMZ. Combination of ILO and TMZ synergistically (CDI < 1) inhibits the growth of U-87 MG cells at a dose of 60 µM. Each bar represents mean ± S.E.M of 3 independent experiments performed. Significant difference is represented by *, where * indicates *p* < 0.05, ** represent *p* < 0.01 and *** indicates *p* < 0.001. (**D**) Photomicrographs (10×) representing 48 h treatment effects of VC, ILO 60 µM, TMZ 60 µM, and their combination on the morphology of U-87 MG cells via phase contrast microscopy. Obvious changes in cellular morphology are observed where cells start to lose their connections, shrink, and become rounded. Arrowheads indicate changes in the cell.

To analyze the growth-inhibitory effect of the ILO and TMZ on GBM cells, U-87 MG cells were exposed to various doses (20 µM, 40 µM, 60 µM, and 80 µM) of these drugs for 48 h, and an MTT assay was performed. The results revealed that both ILO (*p* < 0.05) and TMZ (*p* < 0.01) significantly enhanced the growth inhibition of U-87 MG cells as compared to cells in the vehicle control (VC) group. Also, the inhibition was dose-dependent in the case of ILO treatment. However, no significant difference in growth inhibition was observed between the consecutive dose groups (20 µM, 40 µM, 60 µM, and 80 µM) in the case of TMZ, as revealed by Bonferroni’s post hoc test (Figure 1A,B). 

### 3.2. ILO Acts in Synergism with TMZ against U-87 MG Cells 

U-87 MG cells were treated with various combinations of the test drug ILO with TMZ for 48 h, followed by an MTT assay, to ascertain whether the growth-inhibitory action of TMZ is increased by ILO. After data analysis, it was discovered that, compared to the mono drug treatment, the combination of TMZ 60 µM and ILO 60 µM (ILO + TMZ) significantly improved growth inhibition (*p* < 0.0001) (Figure 1C). In order to determine if the combined effects of TMZ 60 µM and ILO 60 µM were synergistic, additive, or antagonistic, coefficient of drug interaction (CDI) values were also computed. With a CDI value of 0.82, the combined impact of TMZ 60 µM and 60 µM of ILO was found to be synergistic (Figure 1C). For all subsequent experiments, these synergistic doses of drugs were used. The data on the effect of other combinations of ILO and TMZ on the growth inhibition of U-87 MG cells are provided in the Supplementary Materials in Figure S1. To confirm that ILO enhances TMZ activity, we repeated the MTT assay in another GBM cell line, T-98G. Both ILO and TMZ alone significantly inhibited the growth of T-98G cells. However, the effect of ILO was much more pronounced than TMZ, which was also reflected in the combination treatment group, where the growth inhibition was significantly higher in comparison to VC and TMZ alone. However, the results were not synergistic (Figure S2).

### 3.3. ILO, TMZ, and Their Combination Induced Morphological Variations in U-87 MG 

U-87 MG human glioblastoma cells were treated with doses of VC, ILO, TMZ, and their combination for 48 h. The cells were then visualized at 10× magnification under phase contrast microscopy for assessment of the cell morphology. Evident morphological changes were detected among all the treatment groups in comparison to the control; more pronounced changes were observed in the combination group. Predominantly, the cells lost their star-shaped morphology and became rounded. Cell processes and connections with other cells were also lost. Cell shrinking and detachment from the flask surface were also observed (Figure 1D).

### 3.4. Combination of ILO and TMZ Enhanced the Induction of Apoptosis in U-87 MG Cells

**Figure 2:**
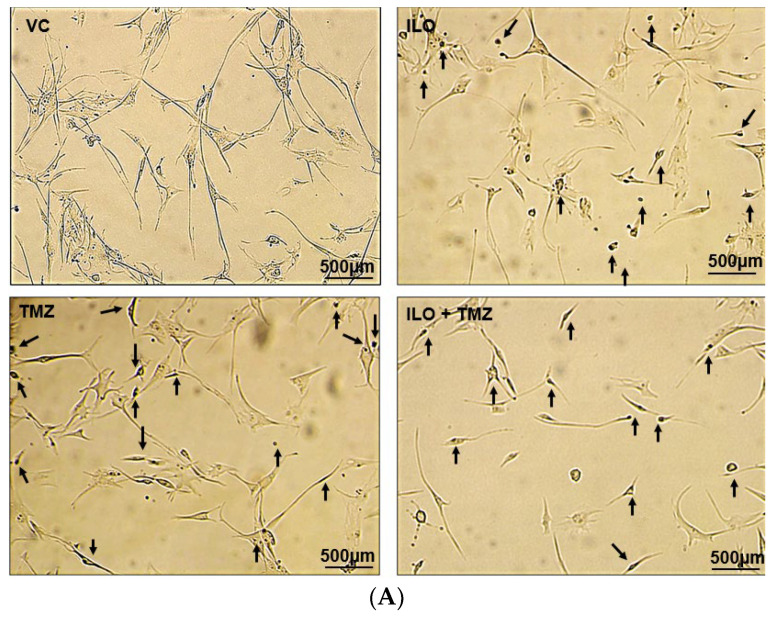

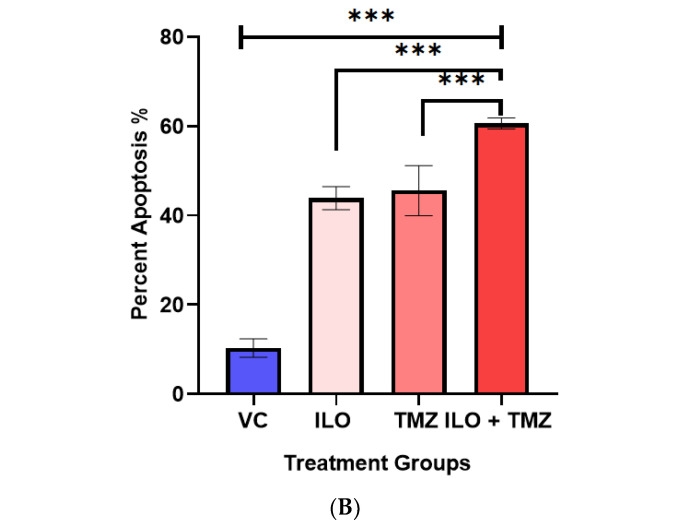
Photomicrographs (**A**) and graphical illustration (**B**) showing induction of apoptosis in treated U-87 MG cells. The U-87 MG cells were exposed to ILO 60 µM, TMZ 60 µM, and their combination for 48 h and observed under an inverted microscope (10×). Dark brown stained cells are symbolic of apoptosis in the above images, which are indicated with arrowheads. Graphical illustrations representing the percentage of apoptotic cells in the case of ILO 60 µM, TMZ 60 µM, and their combination treatment. Data are represented as mean ± S.E.M of 3 independent experiments. Apoptosis was significantly increased in all treated groups as compared to VC, and apoptosis was more pronounced in the combination treatment group. Significant difference is represented by *** which indicates *p* < 0.0001.

U-87 MG cells were treated with VC and selected doses of ILO, TMZ, and their combination for 48 h. To analyze apoptosis, a TUNEL assay was employed. Photomicrographs of the cells were captured under an inverted microscope at 10× magnification. The cells exhibiting apoptosis were visualized as dark brown stained nuclei, indicating DNA fragmentation and cell death by apoptosis (Figure 2A). All the treatment groups induced significant apoptosis as compared to VC (*p* < 0.001). The combination of ILO 60 µM and TMZ 60 µM (ILO + TMZ) also significantly enhanced apoptosis (60.57% ± 1.24%) as compared to individual treatment groups of ILO (34.00% ± 2.58%) and TMZ (40.5% ± 12.4%), as evident by more TUNEL-positive cells (Figure 2B).

### 3.5. ILO Inhibits Migration of GBM Cells When Combined with TMZ

**Figure 3:**
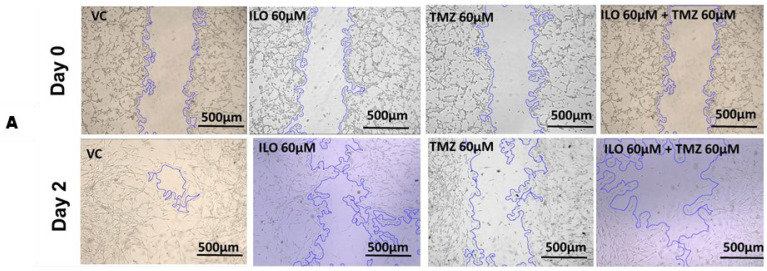

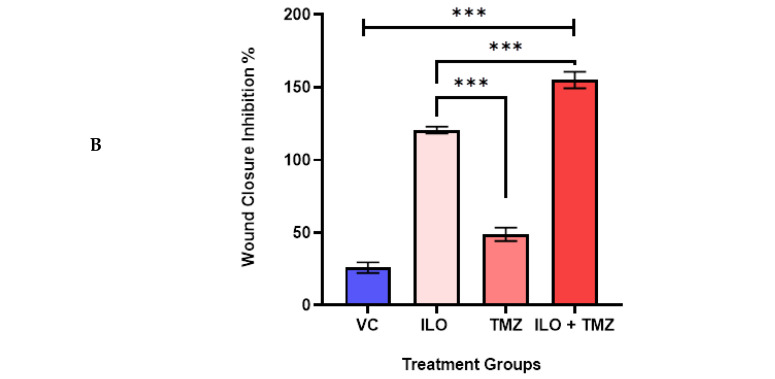
ILO photomicrographs (**A**) and graphical illustration (**B**) showing the effect of treatment of ILO, TMZ, and their combination on cell migration: photomicrographs (10×) showing inhibition of migration of U-87MG cells after 48 h of treatment with ILO 60 µM, TMZ 60 µM, and the combination. A wider scratch area can be seen in the treated group compared to the control. Bar graph showing % wound closure inhibition. Each bar represents mean ± S.E.M of 3 independent experiments. A significant difference was observed in wound closure inhibition % in treated groups as compared to VC, with a more pronounced effect in the combination treatment group. Significant difference is represented by *** which indicates *p* < 0.0001.

To investigate the effect of the drugs on the migration potential of U-87 MG, a wound healing/migration assay was employed. The wound closure area was observed in all treatment groups, and photomicrographs were taken at 4× magnification (Figure 3A). Wound closure area was computed using ImageJ software, and upon statistical analysis, it was revealed that the inhibition of migration is significantly (*p* < 0.0001) pronounced in the combination treatment group (39.80% ± 1.56%) as compared to monotreatment groups of ILO 60 µM (23.62% ± 1.03%) and TMZ 60 µM (12.54% ± 0.48%) (Figure 3B).

### 3.6. ILO Inhibits Invasion of U-87MG Cells When Combined with TMZ

**Figure 4:**
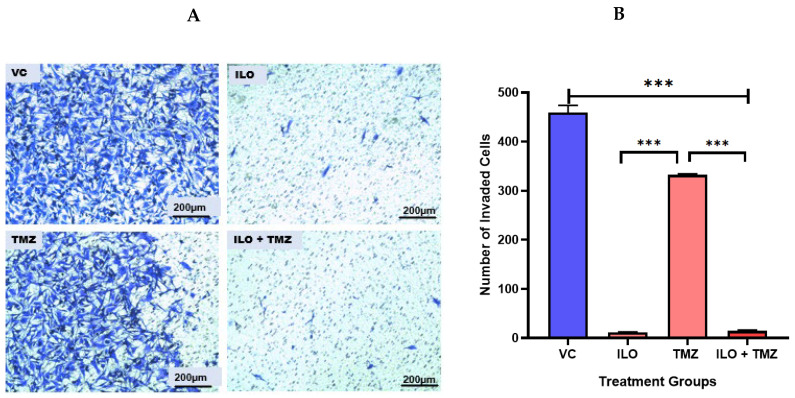
Effect of ILO, TMZ, and their combination on the invasion of U-87 MG cells after 48 h treatment. (**A**) The representative photomicrograph shows that all treatment groups significantly inhibited the invasion of cells in comparison to VC. The number of invasive cells is minimal in the ILO and combination (ILO+TMZ)-treated group in comparison to VC and TMZ. (**B**) Bar graph showing the number of cells that invaded the membrane in different treatment groups. Each bar represents mean ± S.E.M of three independent experiments. Significant difference is represented by *** which indicates *p* < 0.001.

A Transwell Invasion Assay was performed to observe the invasive potential of U-87 MG after a 48 h treatment with ILO, TMZ, and their combination. After treatment, an invasion assay was performed. ImageJ software was used to quantify the invaded cells. Photomicrographs were taken at 10× magnification. Results revealed that all treatment groups significantly (*p* < 0.001) inhibited GBM cell invasion as compared to the control (*p* < 0.001). Further, the number of invasive cells was minimal (*p* < 0.001) in the ILO and combination treatment groups (Figure 4A,B). 

### 3.7. The Expression Analysis of DRD2 and Selected Wnt Pathway Genes

As ILO is an atypical anti-psychotic which works as a DRD2 antagonist, we explored the effect of ILO treatment on DRD2 expression. Further, as mentioned in the introduction, the DRD2 antagonist can modulate the wnt signaling pathway, so we further explored the effect of ILO alone and in combination with the standard drug TMZ on the expression of genes related to this pathway, e.g., β-catenin, Dvl2, Twist, and Slug. 

#### 3.7.1. ILO Downregulated the Expression of DRD2 

**Figure 5:**
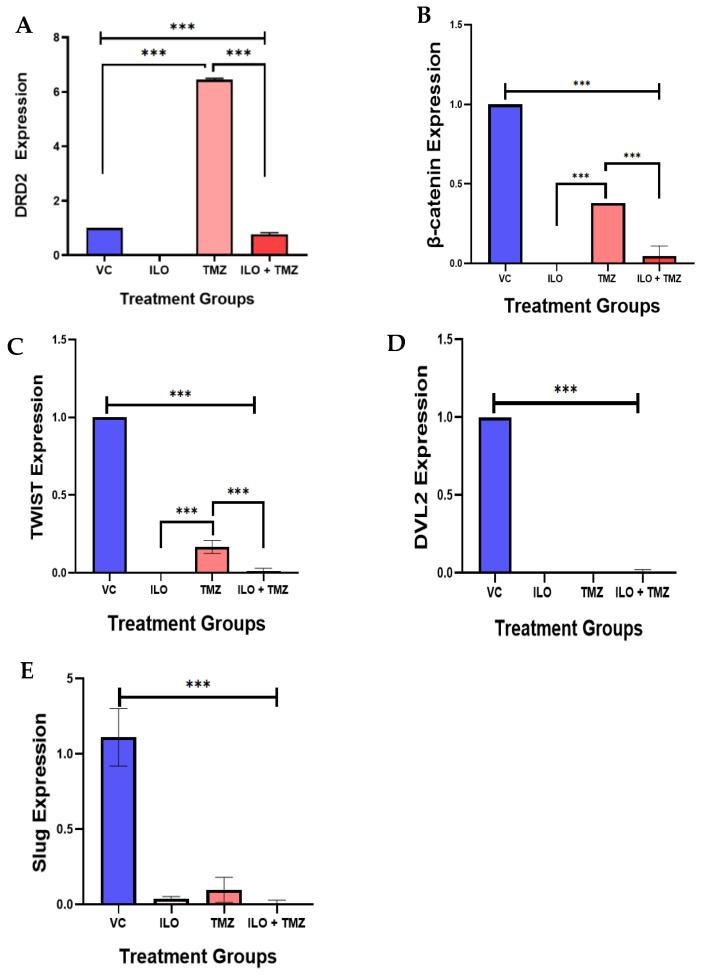
The effect of ILO, TMZ, and their combination on the mRNA expression of the Wnt/β-catenin pathway-related genes (β-catenin, Dvl2, Twist, and Slug) in U-87 MG cells after 48 h of treatment. (**A**) ILO and combination treatment significantly reduced the expression of DRD2 as compared to control, while TMZ upregulated the expression significantly. (**B**) β-catenin expression was significantly reduced in all treatment groups in comparison to VC. However, TMZ increased the expression in comparison to other groups. (**C**) Twist was significantly reduced in all treatment groups in comparison to VC. However, the TMZ-treated cells have a higher expression in comparison to other groups. (**D**) Dvl2 was also significantly reduced in all the treatment groups as compared to the VC group. (**E**) Slug expression was reduced in all the treatment groups compared to the VC. The expression of each gene is normalized with β-actin expression. Each bar represents mean ± S.E.M of three independent experiments. Significant differences between VC and other treated groups were indicated by *** *p* < 0.001.

DRD2 expression was also evaluated in GBM cells after treatment with ILO 60 µM, TMZ 60 µM, and their combination (ILO + TMZ). Quantitative real-time PCR showed that after being treated with TMZ alone, DRD2 expression was significantly upregulated (*p* < 0.001) in comparison to VC and ILO while treatment with ILO significantly (*p* < 0.001) inhibited DRD2 expression as compared to VC and TMZ. This inhibition was also significant in the combination treatment group (*p* < 0.001) as compared to not only VC but TMZ-treated cells as well (Figure 5A).

#### 3.7.2. ILO, TMZ, and Their Combination Downregulated the mRNA Expression of β-Catenin, Dvl2, Twist, and Slug

We observed β-catenin expression in U-87 MG cells after 48 h treatment with ILO, TMZ alone, and in combination. Quantitative real-time PCR demonstrated that β-catenin expression was significantly reduced in all treatment groups (*p* < 0.001) in comparison to VC (Figure 5B). Additionally, no significant downregulation of β-catenin was observed between the treatment groups as revealed by the post hoc Bonferroni test. We observed the expression of transcription factors (Twist and Slug) that regulate the Wnt pathway by interacting with β-catenin. Both Twist and Slug were significantly downregulated in all treatment groups as compared to the control (*p* < 0.001) (Figure 5C,D). 

The post hoc Bonferroni test revealed further significant downregulation of Twist in the combination group as compared to the TMZ alone group (Figure 5C). Dvl2 also regulates the Wnt signaling by modulating GSK-3β, allowing for the nuclear translocation of β-catenin and subsequent Wnt–target gene activation. The expression of Dvl2 was also significantly (*p* < 0.00) reduced in all the treatment groups as compared to the VC group (Figure 5E).

### 3.8. Downregulation of β-Catenin Protein Expression upon Treatment with ILO, TMZ, and Their Combination

**Figure 6:**
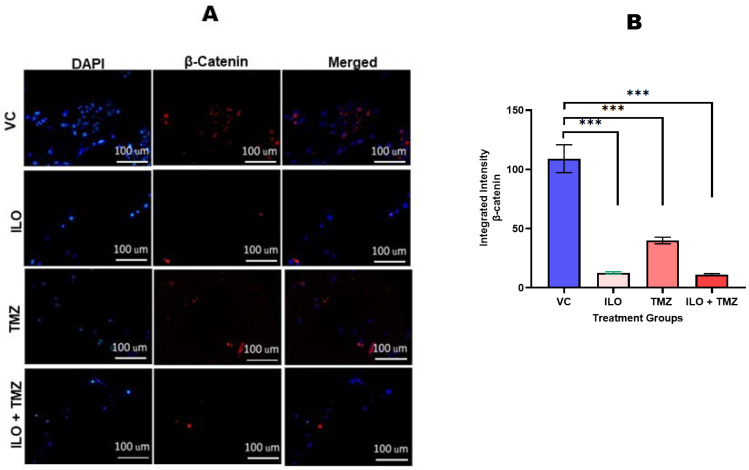
Photomicrographs (**A**) and graphical illustration (**B**) showing the effect of ILO, TMZ, and their combination on β-catenin protein expression. The U-87 MG cells were treated with ILO and TMZ, and the combination of both drugs and immunostaining was performed after 48 h. The images were taken by fluorescence microscope at 20× magnification. The intensity of β-catenin protein expression was analyzed using ImageJ software (Version 1.8.0). Images evidently show the downregulation of β-catenin protein expression in all treatment groups as compared to the vehicle control. Each bar represents mean ± S.E.M of three independent experiments. Significant difference in expression between vehicle control and treated cells is represented by *** which indicates *p* < 0.001.

To confirm the effect of ILO, TMZ, and their combination on β-catenin expression, immunocytochemistry was performed. The mono- and combined drug treatment downregulated the expression of β-catenin, as can be seen in the images obtained using a fluorescent microscope at 10× magnification (Figure 6A). β-catenin fluorescence intensity was measured using ImageJ software (Version 1.8.0) (Figure 6B). β-catenin expression was reduced significantly in all treated groups as compared to the VC (*p* < 0.001). Relatively, the ILO and combination treatment showed a greater inhibition of expression as compared to TMZ. 

## 4. Discussion

The quest of exploring potential therapeutics for GBM is still unanswered despite aggressive research over decades. Current GBM management results in suboptimal clinical outcomes. Drug repurposing offers a safer alternative, allowing faster and inexpensive delivery from bench to bedside. Several drugs have been tried to be repurposed against GBM [1,15,46]. Among these, anti-psychotics have demonstrated strong potential in inhibiting cancer cells [7,38]. They are already used as an adjuvant treatment for cancer-related psychological problems in all cancers, including GBM. Studies have shown a lower incidence of GBM in patients using them [7,15,47].

It has already been established that atypical anti-psychotics are clinically better than typical anti-psychotics but are still underexplored for their anticancer effect in GBM. Therefore, in the present study, we explored the potential of FDA-approved atypical anti-psychotic Iloperidone (ILO) against GBM. Here, we report the in vitro effects of ILO alone and in combination with TMZ on the growth inhibition, apoptosis, and migration of U-87 MG cells. We also analyzed the expressions of DRD2, β-catenin, Dvl2, Twist, and Slug to explore the underlying molecular mechanism of action of ILO and TMZ alone and in combination. 

ILO and TMZ inhibited the growth of U-87 MG cells significantly when used alone. Interestingly, ILO inhibited GBM cells in a dose-dependent manner, as we noticed a better effect when the dose was increased. The results are comparable to a study by Varalda et al., according to which ILO inhibited U-87 MG cells at IC_50_ of 80 µM [38], but the study did not explore ILO for further mechanistic activity. We did not observe any dose-dependent effects in TMZ-treated cells, although some studies have reported it [42,44]. This could be because those studies have used a TMZ dose of more than 100 µM to observe its cytotoxic effect [48], while our test dose ranged from 20 µM to 80 µM. Next, the combined effect of both the drugs was observed in U-87 MG and T-98G, and it was noted that ILO synergizes with TMZ to inhibit the growth of U-87 MG cells as the CDI value was found to be less than 1. Anti-psychotics like chlorpromazine [16,49], trifluoperazine [50], pimozide [51], and olanzapine [19] have also shown growth inhibition and apoptotic activity in different types of cancers/cell lines. In the case of T-98G, while the effect of the co-treatment of drugs was higher as compared to VC and TMZ alone, the results were not synergistic, which may be because of the resistance of T-98G cells to TMZ, as these cells have higher expression of MGMT, one of the TMZ resistance factor [4,52]. To determine if the growth inhibition involves cell death via apoptosis, apoptotic morphology was observed in all treatment groups along with DNA fragmentation using a TUNEL assay that can identify nicks in the DNA. Induction of apoptosis was more pronounced in the combination treatment group as compared to mono-treatment by ILO and TMZ. The results are in accordance with the studies that have explored the apoptotic potential of other anti-psychotics like haloperidol, risperidone, and olanzapine and have found that DRD2 antagonists enhance the apoptotic activity of TMZ [10,12,53]. Consequently, it can be inferred that the growth inhibitory activity of ILO involves apoptosis, and it can also enhance the apoptotic potential of TMZ. 

A wound healing and invasion assay was used to observe if ILO, TMZ, and their combination inhibit the migration and invasion of U-87 MG cells. Cell migration was reduced in all treatment groups; however, the combination of ILO and TMZ showed the greatest effects. This is contrary to the study where it was observed that Olanzapine alone and in combination had no effect on the migratory potential of U-87 MG but had an anti-migratory effect on the A172 cell line [19]. ILO, TMZ, and their combination showed significant effects on the inhibition of invading glioma cells. Chlorpromazine, which is a typical anti-psychotic, has been shown to inhibit the invasion of glioma cells alone and in combination with TMZ [54]. Likewise, Trifluoperazine, another typical anti-psychotic, also inhibited cell proliferation, migration, and the invasion of U-87 MG cells [55].

Further, we hypothesized that the synergistic activity of ILO with TMZ might be due to its effect on DRD2 receptors as, according to many studies, the overexpression of DRD2 is associated with the progression of many cancers, including GBM [8,10,48]. Therefore, we analyzed DRD2 expression in all treatment groups and found significant overexpression of DRD2 in the TMZ-treated group in comparison to the control. The upregulation of DRD2 in the TMZ-treated group could be one of the bases of limited activity of TMZ in GBM [10,12,53]. Interestingly, DRD2 antagonist, ILO alone, and in combination with TMZ significantly inhibited the expression. This clearly signifies that ILO, a DRD2 antagonist, suppresses DRD2 expression upregulated by TMZ, creating an effective milieu. 

Besides the dopamine pathway, it has also been reported that DRD2 antagonists modulate the Wnt pathway via GSK-3β. GSK-3β forms a complex along with APC that is responsible for phosphorylating β-catenin, targeting it for destruction [23,56]. The Wnt pathway is found to be overexpressed in the majority of cancers, including GBM, resulting in their proliferation and progression [26]. Therefore, we also investigated the effect of treatment on Wnt pathway genes via β-catenin, Dvl2, and genes modulated by the β-catenin pathway, including Twist and Slug. 

β-catenin expression was investigated in all treatment groups, and it was observed that the expression reduced significantly at both RNA and protein levels in comparison to VC. Upregulation in β-catenin expression was observed in the TMZ-treated group in some studies, which is contrary to our results. The reason could be the use of a higher dose (100–500 µM) in comparison to the TMZ dose used in the present study [26]. As β-catenin expression was significantly higher in the TMZ-treated group in comparison to the ILO-treated and combination-treated groups, it can be inferred that significant downregulation in β-catenin expression in the combination-treated group can be an additional factor in enhancing TMZ activity while reduced β-catenin expression in ILO treated group may be because of its inhibitory activity on DRD2. As studies have shown, DRD2 antagonists inhibit the Wnt pathway via GSK-3β, leading to the proteasomal degradation of β-catenin and hence its downregulation [57,58].

Next, we explored the expression of Dvl2 and found significantly reduced expression of Dvl2 in all treatment groups as compared to the control, indicating ILO, TMZ, and their combination equally inhibit Dvl2, limiting cancer progression. Dvl2 proteins are located in the cytoplasm and are pivotal in the transduction of Wnt signaling from the receptor level to the intracellular compartment. It regulates the Wnt signal by modulating GSK-3β, allowing nuclear translocation of β-catenin and subsequent Wnt-target gene activation. It has also been found to be overexpressed in GBM [8,36,59].

Moreover, we investigated the expression of transcription factors that have previously been reported to be associated with the invasion and migration of GBM cells. In our study, the expressions of Twist and Slug were significantly downregulated in all treatment groups as compared to the control. The significant effect of these treatments on the migration of U-87 MG cells might be due to the inhibition of these two genes as both Twist and Slug regulate epithelial–mesenchymal transition and are associated with invasion, migration, and hence, therapeutic resistance in GBM [7,8]. 

In summary, our investigation found that using ILO and TMZ led to a decrease in Dvl2 expression. This decrease could result in stabilizing the destruction complex, which consists of APC, axin, and GSK-3β [9]. As a result, β-catenin is targeted for proteasomal degradation and does not migrate into the nucleus. Consequently, this action turns off the Wnt signaling pathway, leading to a reduction in Twist and Slug expression (Figure S3). 

## 5. Conclusions

In conclusion, we here provided evidence that Iloperidone demonstrates anti-proliferative and pro-apoptotic activity in GBM. It also synergizes with TMZ, which may be related to its inhibitory effect on DRD2 and β-catenin expression.

## 6. Limitations of the Study

The study explored the mRNA expression of some selected Wnt/ β-catenin signaling genes (β-catenin, Twist, Dvl2, and Slug) as well as the protein expression of only β-catenin while changes in the expression of other pathway proteins in response to the treatment can also be investigated. Secondly, the study is limited to the in vitro approach. An in vivo study can be employed to further confirm the findings.

## Data Availability

Data are contained within the article and Supplementary Materials.

## Supplementary Materials

The following supporting information can be downloaded at: https://www.mdpi.com/article/10.3390/biomedicines12061134/s1, Figure S1: The Graphical Illustrations of the Combined Effects of ILO and TMZ on T-98 G Growth. Figure S2: The effects of ILO, TMZ and their combination on U-87 MG growth inhibition. Figure S3: Illustrative view of the canonical Wingless (WNT)/β-catenin signaling and proposed Iloperidone mechanism via Wnt signaling. Table S1: List of Primer sequences Used Ain the study.
