# Supplementary material for: Iloperidone and Temozolomide Synergistically Inhibit Growth, Migration and Enhance Apoptosis in Glioblastoma Cells
Source: Biomedicines. 2024 May 21;12(6):1134. doi: 10.3390/biomedicines12061134 (PMC11200733; doi:10.3390/biomedicines12061134)
Supplement: Supplementary file 1 [file biomedicines-12-01134-s001.zip › biomedicines-2980470-supplementary.pdf]

Supplementary Material

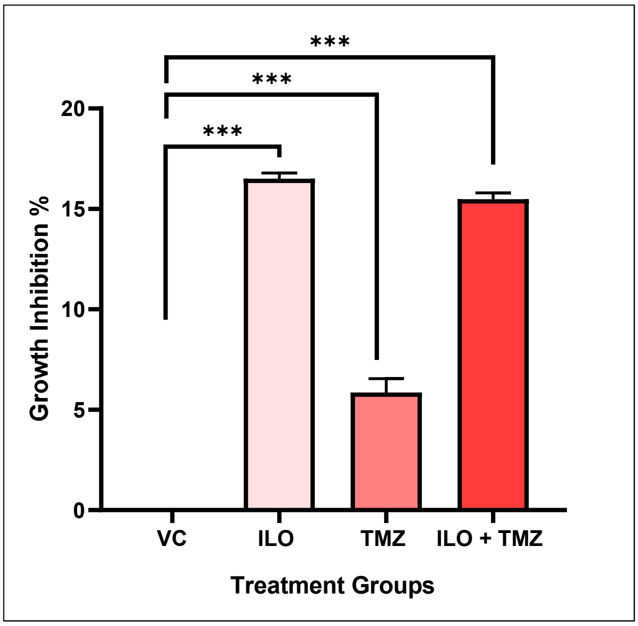

Figure S1. The Graphical Illustrations of the Combined Effects of ILO and TMZ on T-98 G Growth. The cells were treated with ILO (60µM), TMZ (60µM) and their combination for 48 hours followed by MTT assay. All treatment groups significantly inhibited the growth of T-98G cells in comparison to VC. Each bar represents mean ± S.E.M of 3 independent experiments performed. significant difference is represented by *. Where, *** indicates P <0.001.

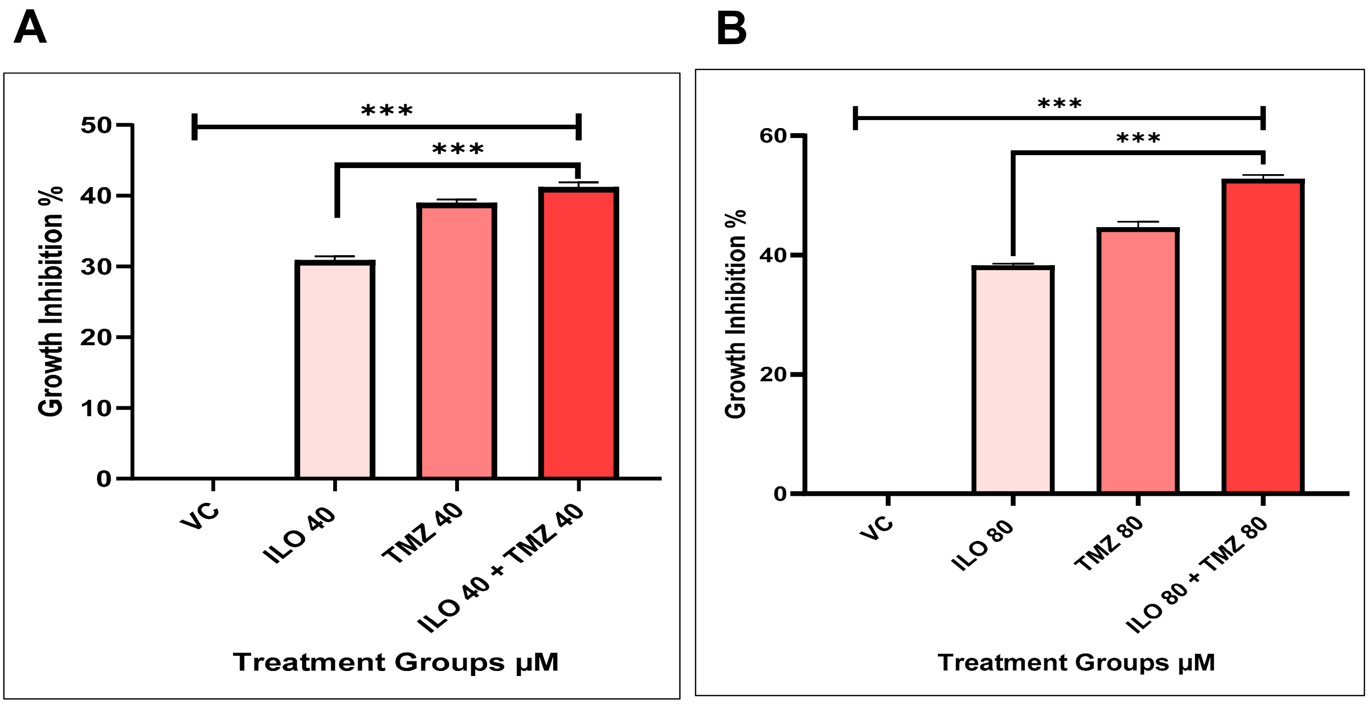

Figure S2: The effects of ILO, TMZ and their combination on U-87 MG growth inhibition. The cells were treated with different doses of ILO and TMZ (40µM and 80µM) and their combination for 48 hours followed by MTT assay. A) Bar graph illustrating significant growth inhibition of U-87 cells with ILO 40µM, TMZ 40µM and ILO+TMZ 40µM+ 40µM in comparison to VC. B) Bar graph illustrating significant growth inhibition of U-87 cells with ILO 80µM, TMZ 80µM and ILO+TMZ 80µM+ 80µM in comparison to VC. Each bar represents mean ± S.E.M of 3 independent experiments performed. *** indicates $P < 0.001$ and significant difference between treatment groups and VC.

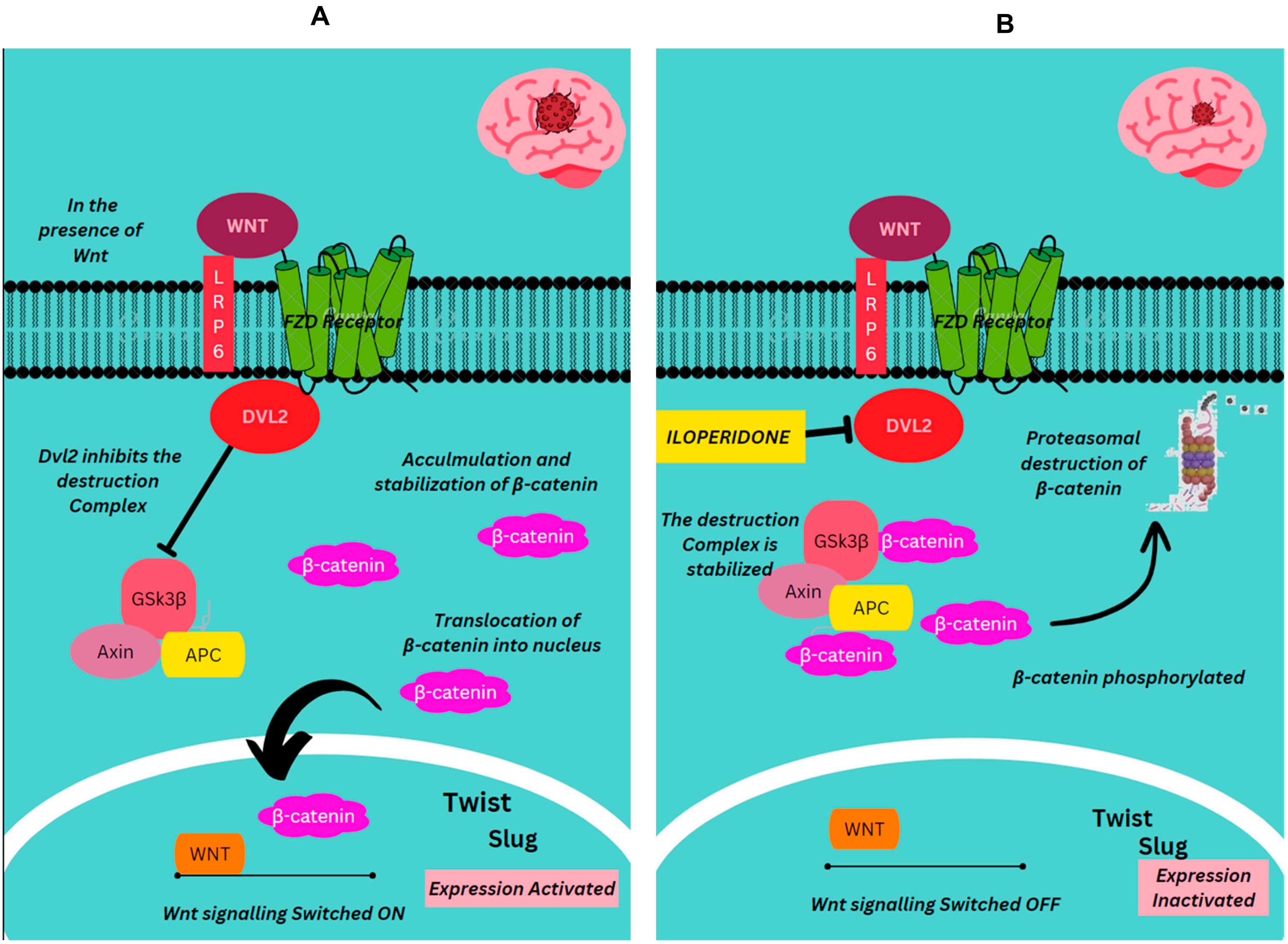

Figure S3: Illustrative view of the canonical Wingless (WNT)/β-catenin signaling and proposed Iloperidone mechanism via Wnt signaling: A) The binding of WNT ligand to frizzled and co-receptors Low-density lipoprotein receptor-related protein 6 (LRP6) activates Disheveled (DVL 2) which leads to the inactivation of the destruction complex (adenomatous polyposis coli (APC), axin and glycogen synthase kinase-3β (GSK-3β). This allows β-catenin to accumulate in the cytoplasm and translocate to the nucleus, where it forms a transcriptionally active complex and activates transcription of transcription factors, Twist, and slug. **B**) Iloperidone potentially inhibits DVL 2 causing stabilization of the destruction complex, and β-catenin is then targeted for proteasomal degradation and therefore cannot enter into the nucleus, causing switching off the wnt signaling pathway and hence, further expression of the Twist and slug. *Adapted from Denysenko et al, 2016,* (57).

Table S1: List of Primer sequences Used in the study.

Primers	Sequence	Annealing Temp °C
**DRD2**	5′-AGACCATGACCGTAGGAAG -3<br>5′- GCAGCCAGCAGATGATGA -3	59
**β Actin**	5′-CACTGGCATCGTGATGGACT -3<br>5′- TGGCCATCTCTTGCTCGAAG -3	58
**β-catenin**	5′-TCTGAGGACAAGCCACAAGATTACA-3′<br>5′TGGGCACCAATATCAAGTCCAA-3′	60
**Twist**	5′-GCAAGAAGTCGAGCGAAGAT-3′<br>5′-GCTCTGCAGCTCCTCGAA-3′	60
**Dvl2**	5′TGAGCAACGATGACGCTGTG-3′<br>5′GCAGGGTCAATTGGCTGGA-3′	62
**Slug**	5′GAGCATTTGCAGACAGGTCA-3′<br>5′CCTCATGTTTGTGCAGGAGA-3′	60.4
